# Automatic vs. rational motives for adolescent physical activity: an ecological momentary assessment study

**DOI:** 10.3389/fpsyg.2026.1727943

**Published:** 2026-05-21

**Authors:** Calissa J. Leslie-Miller, Zachary Bricken, Christopher C. Cushing

**Affiliations:** Department of Clinical Child Psychology/Life Span Institute, University of Kansas, Lawrence, KS, United States

**Keywords:** adolescents, affect, competence, motivation, physical activity, social support

## Abstract

**Objective:**

The current study seeks to examine rational motivational variables central to self-determination theory (i.e., perceived competence, family social support, friend social support, autonomous motivation, controlled motivation, and amotivation) as moderators of the relationship between automatic motives (i.e., momentary affective states, fatigue, and energy) and physical activity (moderate-to-vigorous physical activity and sedentary behavior).

**Methods:**

We conducted a 20-day ecological momentary assessment (EMA) study, in which 100 adolescents aged 13–18 years old were recruited locally February 2016 to March 2017 and were asked to indicate baseline social support, motivation, and competence for physical activity. Subsequently, participants wore an Actigraph accelerometer 24 h per day and answered EMA surveys assessing automatic motives 4 times per day using time-based signaling.

**Results:**

There was a significant interaction between within-person positive affect and amotivation as a predictor of moderate-to-vigorous physical activity (MVPA) (*b* = −0.02, *p* < 0.05) with 95% CI [−0.04, −0.01] and sedentary time (*b* = 0.10, *p* < 0.01) with 95% CI [0.04, 0.16]. There was also a significant interaction between within-person negative affect and amotivation as a predictor of sedentary time (*b* = −0.08, *p* < 0.05) with 95% CI [−0.14, −0.01]. Within-person fatigue interacted with friend social support (*b* = 0.05, *p* < 0.01) with 95% CI [0.02, 0.08], amotivation (*b* = 0.03, *p* < 0.05) with 95% CI [0.003, 0.06], and controlled motivation (*b* = −0.03, *p* < 0.01) with 95% CI [−0.05, −0.01] in relation to MVPA.

**Conclusions:**

Overall, findings from this study suggest that rational and automatic motives are intertwined and should be explored together as targets for physical activity intervention efforts.

## 1 Introduction

Despite the obvious importance of regular physical activity, 81% of adolescents do not meet the best available guidelines for sufficient physical activity (Guthold et al., 2020). To promote overall health and well-being, the US Department of Health and Human Services recommends that adolescents engage in 60-min of moderate-to-vigorous physical activity (MVPA) each day and limit sedentary behavior (Piercy et al., 2018). By adhering to these guidelines, adolescents can effectively manage various health conditions and reduce the risk of negative health consequences (e.g., depression, anxiety, cardiovascular disease, diabetes, cancer; Dishman et al., 2022; Rodriguez-Ayllon et al., 2019). However, despite the significance of these recommendations, studies indicate a decline in physical activity levels among adolescents (Farooq et al., 2020), coupled with an increase in sedentary time (Cooper et al., 2015). In general, low levels of MVPA and high levels of sedentary time cannot be attributed to lack of knowledge about the importance of these behaviors or low capability. Adolescents know that physical activity is important, and so do their parents (Academy, 2008), however this knowledge does not appear to translate to motivation to participate in physical activity. Among individuals who are more likely to engage in physical activity, several motivational factors have been linked to their success, including enjoyment of the activity, health benefits, well-being, and social support (Pedersen et al., 2021). Therefore, motivation seems to be a valuable study target in the hopes of spurring intervention efforts.

Motivation to engage in ideal MVPA and limit sedentary time (hereafter physical activity) can be classified into two main categories. First, rational processes drive engagement through well-studied internal factors, such as the drive for autonomy, a desire for relatedness, and competence, which are central to self-determination theory (SDT). SDT distinguishes between both the satisfaction of basic psychological needs (i.e., autonomy, competence, and relatedness) and the quality of behavioral regulation. Within this framework, individuals may engage in physical activity for more autonomous reasons (e.g., enjoyment), more controlled reasons (e.g., obligation), or may experience amotivation (Buchan et al., 2012). This type of motivation might involve a desire to exercise because a friend is walking in the same park, aiming to achieve a personal best in an athletic event, or seeking a sense of self-direction in choosing leisure activities. Both need-related processes and more self-determined forms of motivation have been linked to increased engagement in physical activity among adolescents. For example, the fulfillment of psychological needs, including autonomy and competence, has been found to positively predict motivation for sport participation, leading to subsequent engagement in physical activity (Sebire et al., 2013). Further, the drive for autonomy and the desire for relatedness have been linked to increased involvement in physical activity for adolescents (Gourlan et al., 2013). Although social support is not a core construct with SDT, it represents an important contextual factor that may facilitate the satisfaction of psychological needs, particularly relatedness (Pedersen et al., 2021).

Taken together, the literature suggests that autonomy, competence, and relatedness each play a role in physical activity, both independently and in combination (Buchan et al., 2012; Gourlan et al., 2013; Sebire et al., 2013). However, it appears that people also engage in suboptimal physical activity for reasons that do not originate from rational thought and instead arise from a second, more automatic, subsystem that occurs outside careful conscious consideration (Brand and Cheval, 2019). Motives that correspond to more automatic and situationally variable inputs are commonly related to subjective feelings such as affect, energy, or fatigue (Brand and Ekkekakis, 2018; Cushing et al., 2017). These feelings enhance or diminish motivation in powerful ways similar to the rational subsystem but may also vary from person-to-person (Dunton et al., 2014). For example, a person may know that they need to exercise to achieve a health goal, be invited to exercise by a loved one with whom they desire connection, and still fail to engage in exercise due to “feeling too tired” (i.e., fatigue). These physical-feeling states have been shown to predict later physical activity and can be observed at the person level (Dunton et al., 2014). The complex nature of these states and physical activity has also been observed as a bidirectional relationship (Cushing et al., 2017), furthering the need to parse the intertwined relationship between them.

As children transition into adolescence and shift toward less structured physical activity, gaining more autonomy in selecting activities, motivational factors become increasingly crucial for sustaining healthy habits. The transition to adolescence may also introduce more stressful environments, wherein stress has been associated with decreased physical activity (Do et al., 2021). An adolescent's self-concept may also impact their motivation to engage in physical activity, particularly among females (Schneider et al., 2008).

Much of the current research on motivational factors for adolescent physical activity exists within contextual feelings of positive and negative affect and physical-feeling states (i.e., energy and fatigue). Generally, higher positive affect and feelings of energy are predictive of higher physical activity, while higher negative affect and feelings of fatigue are predictive of lower physical activity (Dunton et al., 2014); however, our recent work has indicated that high self-efficacy may buffer against the deleterious impacts of negative affect (Leslie-Miller and Cushing, 2024). Affectively charged motivations have been shown to be predictive of physical activity in older populations, where higher positive affect in the morning could assist in encouraging physical activity through the day (Crosley-Lyons et al., 2023). Additionally, given the potential variability of affective states over time and the bi-directional nature of affect and physical-feeling states in relation to physical activity (Cushing et al., 2017; Dunton et al., 2022), assessing additional motivational factors may yield new insights into these dynamic processes. Other theoretical frameworks have similarly sought to integrate rational and automatic processes in the context of physical activity, such as the Integrated Behavior Change Model (Hagger and Chatzisarantis, 2014), which emphasizes the role of both reflective and automatic influences on behavior. Despite growing evidence for both affective and cognitive motivational systems, few studies have examined how these processes interact to influence adolescent physical activity.

With a large body of research on factors associated with adolescent physical activity, continued work in identifying additional motivators remains an important area of study. The current study seeks to explore the association of cognitively effortful processes and automatic processes as motivators of adolescent physical activity. Currently, the literature on this topic is largely divided into studies of rational motives for physical activity in one set of articles, and automatic motives in another literature. It is indeed quite likely that the two processes are intertwined such that some rational processes may help to overcome vulnerabilities to poor physical activity presented by automatic processes such as affect, fatigue or energy. Adolescence is a key period of development when self-regulation of affect, cognition, and behavior is not yet fully formed. Consequentially, adolescents should be particularly vulnerable to changes in the automatic subsystem that exert a negative impact on physical activity. Therefore, the goal of the current project was to determine whether automatic vulnerabilities to low physical activity (i.e., low MVPA and high sedentary behavior) can be overcome through effortful, conscious reasoning. Accordingly, the primary goal of the current project was to test whether rational and automatic motivational processes interact in predicting physical activity. We hypothesized that automatic processes (e.g., affective states, fatigue, and energy) would be associated with momentary MVPA and sedentary behavior, and that these associations would be moderated by rational motivational factors (i.e., perceived competence, family social support, friend social support, autonomous motivation, controlled motivation, and amotivation).

## 2 Method

### 2.1 Participants

One-hundred adolescents aged 13–18 years old (*M* = 14.45, SD = 1.37) were recruited locally from February 2016 to March 2017 throughout the community in a mid-sized midwestern college town. Participants were recruited to take part in a 20-day ecological momentary assessment (EMA) study in which they were asked to complete a baseline assessment of their physical activity, perceived competence, social support, and motivation. Subsequently, participants wore an Actigraph accelerometer 24 h per day and answered EMA surveys assessing affect four times per day using time-based signaling. Adolescents were eligible to participate if they were between the ages of 13 and 18 years old, did not have any significant visual impairments, lived at home with their parents, did not have any significant physical maladies that would limit physical activity, and were able to read at grade level in English.

### 2.2 Materials

#### 2.2.1 Moderate to vigorous physical activity and sedentary behavior

The ActiGraph wActi Sleep-BT accelerometer (ActiGraph LLC, Pensacola, FL) was worn on the non-dominant wrist and used to objectively measure MVPA and sedentary behavior. Data were collected via 1 s epochs at a sampling rate of 30 Hz and were aggregated to 60 s using the Actilife software v.6.10.2 (Actilife software v.6.10.2, Pensacola, Florida, Unites States). The Troiano algorithm (TActiGraph, n.d.) was used to define a non-wear period, such that the minimum length was set to 60 min, the spike tolerance was set to 2 min, and the spike level to stop was set to 100 counts per minute. Additionally, the minimum wear time per day was set to 600 min and all sleep periods were marked as non-wear time. According to the Chandler algorithm (Chandler et al., 2016), physical activity data were separated into three categories: sedentary, light, and MVPA, with cut points of sedentary (0–3,660), light activity (3,361–9,804), and MVPA (9,805 and above). The number of minutes of MVPA and sedentary behavior 30-min following each EMA prompt were the data of particular interest for this study. This 30-min window was selected to align with prior EMA research on physical activity (e.g., Dunton et al., 2014). MVPA and sedentary behavior were calculated by summing activity within the 30 min following each EMA survey, which were linked via electronic time stamps.

#### 2.2.2 Positive and negative incidental affect

The Positive and Negative Affect Schedule for Children (PANAS-C; Ebesutani et al., 2012) was used to measure positive and negative affect. This questionnaire consists of 10 self-report items, with half measuring positive affect (i.e., joyful, cheerful, happy, lively, proud; α = 0.94) and half measuring negative affect (i.e., miserable, mad, afraid, scared, sad; α = 0.85). Participants were asked to rate the extent to which they currently feel each affective state on a scale of one (not at all) to five (extremely).

#### 2.2.3 Energy and fatigue

The Profile of Mood States (POMS) was used to assess energy and fatigue (McNair et al., 1971). The three highest loading items for the “Vigor-Activity” (i.e., energetic, full of pep, and vigorous; α = 0.86) and the “Fatigue-Inertia” (i.e., fatigued, exhausted, and worn out; α = 0.88) were used to measure energy and fatigue, respectively. Participants were asked how much they currently felt each physical feeling state since the last prompt on a scale of one (not at all) to five (extremely).

#### 2.2.4 Social support for exercise

The social support for exercise questionnaire (Sallis et al., 1987) was used to assess the degree to which family members and friends provide support for physical activity engagement. The questionnaire consists of 13-items pertaining to support from family (α = 0.84) and 13-items pertaining to support from friends (α = 0.92).

#### 2.2.5 Motivation

The Treatment Self-Regulation Questionnaire (Levesque et al., 2007) assess motivation for engaging in healthy physical activity. The questionnaire consists of 15-items that assess amotivation (*a* = 0.69), autonomous motivation (*a* = 0.90), and controlled motivation (*a* = 0.82) for engaging in physical activity.

#### 2.2.6 Perceived competence scale

The Perceived Competence Scale (Williams et al., 1998) is a four-item measure assessing competence as a motive for physical activity, which is one of the core motives for SDT (α = 0.93). The measure was scored on a 1–7 scale with anchors at one, four, and seven. An example item is,

“*I am able to meet the challenge of exercising regularly.”*

### 2.3 Procedures

Parents and adolescents who expressed interest in participating were instructed to contact the laboratory for a phone screening to assess their eligibility. If a potential participant was deemed eligible but under 18 years of age, they were requested to provide a phone number for a parent or guardian. Study staff would then contact the parent or guardian to provide an explanation of the project and schedule an in-person visit. If the potential participant was eligible and over 18 years of age, or if the call was made by the parent, then the in-person visit was scheduled during the telephone screening. All procedures of the study received approval from the local Institutional Review Board. Prior to engaging in any part of the study, parental consent was obtained and participants under 18 provided assent.

During the initial in-person session, participants were asked to complete a baseline assessment which included measurement of their physical activity, perceived competence, social support, and motivation, and demographic information. They were also given an Actigraph (ActiGraph LLC, Pensacola, FL) and were instructed to wear the monitor on their non-dominant wrist. Since the monitor was waterproof and did not need to be charged during the 20-day study period, participants were asked to wear the monitor 24/7 for the duration of the study. Participants were provided with an Android Smartphone to answer questionnaires, each study day. Participants self-selected four times that would consistently be the most convenient time for them, weekdays, and weekends. Each time selected was required to be two hours apart from each other and participants were recommended to pick two morning times and two afternoon/evening times. After the 20-day study period concluded, participants had an in-person session where they returned the equipment and were given compensation. Participants were paid on a progressive payment schedule such that they earned $0.75 each time they were compliant with study sensors for 12 h a day and a $25 bonus for completing all four of the surveys on 85% of the study days, with total potential compensation of $40.

### 2.4 Data analysis

Descriptive statistics were run using IBM SPSS Statistics (Version 28, Armonk, New York, United States) for all study variables. To assess significant predictors of MVPA and sedentary behavior, multilevel models were run using SAS PROC MIXED (SAS Version 9.4, Cary, NC, United States) to account for the nested data structure: observation of data (level one) nested within a person (level two). We analyzed patterns of missingness at the prompt level using a generalized linear mixed model and found no evidence that study timing (day, prompt number, time of day) or contextual factors (school day, weekend) were associated with the likelihood of missing responses. Following best practices for handling data assumed to be missing at random, full maximum likelihood estimation was used to account for missing data for all models (Enders and Bandalos, 2001). First, we computed intraclass correlation coefficients (ICC) to determine the proportion of variance of the physical activity variable and the sedentary time variable that is within-person or between-person. Next, model comparisons were made for a fixed linear, random linear, fixed quadratic, random quadratic, fixed cubic, and random cubic effect of time, where time was defined as measurement occasion (i.e., each EMA prompt across the study period). For moderate to vigorous physical activity, time was modeled as a fixed linear effect and for sedentary behavior, time was modeled as a random linear effect after testing and rejecting other time trajectories.

Separate models were run for positive affect, negative affect, fatigue, and energy, which were added as between-person and within-person components. Level-one (within-person) variables (i.e., affect, fatigue, energy) were specified with random slopes to allow their associations to vary across individuals when this improved model fit. The between-person component consisted of a person's mean affect across all observations, while the within-person component was created by person-centering the affect variable, that is subtracting the value at each observation from the person's mean across all observations (Raudenbush and Bryk, 2002). Using this centering procedure, between and within variance components are orthogonal to each other and can be included in the same model. Each hypothesized level two predictors (perceived competence, family social support, friend social support, autonomous motivation, controlled motivation, and amotivation) were added to the model independently and evaluated for significance. Significant predictors were retained for final models.

When models were nested, model comparisons of the –Two Restricted Log Likelihood were made and when models were not nested, the Akaike Information Criterion was used to assess model fit.

## 3 Results

Data for 6,947 observations were analyzed, with each participant completing an average of 42 observations (SD = 26.87). The sample was 60% female and 80% White, with 78% of participants reporting their family income as $51,000 or above (Table 1). Prior to parsing any variance components, scores for variables were as follows: positive affect (*M* = 15.02, SD = 5.30), negative affect (*M* = 5.98, SD = 3.29), fatigue (*M* = 2.49, SD = 2.77), energy (*M* = 4.05, SD = 3.28), perceived competence (*M* = 4.29, SD = 1.40), friend social support (*M* = 2.15, SD = 1.03), family social support (*M* = 2.23, SD = 0.77), autonomous motivation (*M* = 5.02, SD = 1.24), controlled motivation (*M* = 3.06, SD = 1.38), and amotivation (*M* = 1.85, SD = 1.13).

**Table 1:** Baseline demographic characteristics.

Baseline characteristic	*N*
Sex	
Female	60
Male	39
Other	1
Total	100
Age	
13	31
14	26
15	23
16	10
17	7
18	3
Race	
White	80
Black	3
Asian	4
Latino/Latina	7
American Indian	1
Multiracial	2
Other	2
Missing	1
Total	100
Family Income	
<$10,000	1
$10,000–$20,000	5
$21,000–$30,000	9
$41,000–$50,000	2
$51,000–$60,000	19
>$60,000	59
Missing	1
Total	100

### 3.1 Moderate to vigorous physical activity

#### 3.1.1 ICC

The results of the null model demonstrated that 7% of the variance in MVPA engagement was between-person. Importantly, this means that 93% of the variance can be accounted for by within-person moment-to-moment changes.

#### 3.1.2 Positive affect

In the final model (Table 2), between-person positive affect and amotivation did not significantly predict engagement in MVPA. However, there was a significant main effect of within-person positive affect (β = 0.06, *p* < 0.01) with 95% CI [0.03, 0.10], and a significant interaction with amotivation (β = −0.02, *p* < 0.05) with 95% CI [−0.04, −0.01].

**Table 2:** MVPA: results for positive affect.

Fixed effects		95% CI for slope		
	Slope	Lower	Upper	*P*
Intercept	0.54	−0.14	1.22	0.12
Time	0.00	−0.00	0.00	0.33
BP positive affect	−0.01	−0.05	0.03	0.69
WP positive affect	0.06	0.03	0.10	**0.001**
Amotivation	−0.09	−0.23	0.05	0.19
WP positive affect^*^amotivation	−0.02	−0.04	−0.01	**0.01**
Perceived competence	0.15	0.03	0.27	**0.02**
Random effects	Estimate		SE	
Intercept	0.43		0.08	
WP positive affect	0.00		0.00	
Residual	5.87		0.11	

BP, between-person; WP, within-person; CI, confidence intervals.

Bold values indicate statistically significant results (*p* < 0.05).

**Figure 1:**
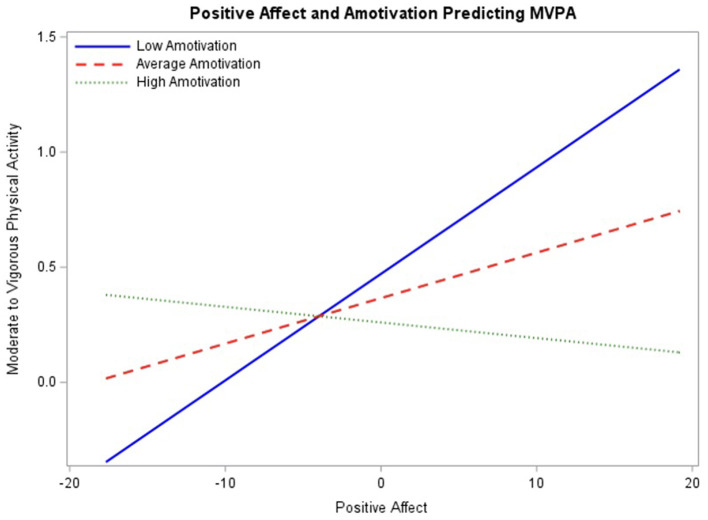
Interaction between positive affect and baseline amotivation predicting engagement in physical activity probed at the mean level of motivation, as well as one standard deviation above and below the mean.

To assess the association between positive affect and engagement in MVPA throughout the range of baseline amotivation ratings, the interaction was probed at mean amotivation ratings as well as one standard deviation above and below the mean (Figure 1). At lower levels of amotivation, the simple slope between positive affect and engagement in MVPA, 0.05, was significant (*p* < 0.01). The simple slope was also significant at the mean of amotivation, 0.02 (*p* < 0.05). At one standard deviation above the mean, the simple slope, −0.01, was not significant (*p* > 0.05). For average and lower levels of amotivation, as within-person positive affect increased, engagement in MVPA increased. Regions of significance testing demonstrated interaction significance between positive affect and MVPA beyond 1.91 for low amotivation and 5.69 for high amotivation. Additionally, there was a significant main effect of perceived competence (β = 0.15, *p* < 0.05) with 95% CI [0.03, 0.27]. This model suggests that when amotivation is low, higher-than-usual positive affect is associated with engagement in MVPA, but this effect diminishes and disappears as amotivation increases. Independent of amotivation, perceived competence has an effect on MVPA.

#### 3.1.3 Negative affect

In the final model (Table 3), within-person negative affect, perceived competence, and autonomous motivation did not significantly predict engagement in MVPA. The only predictor that remained significant was between-person negative affect (β = 0.08, *p* < 0.05) with 95% CI [0.01, 0.16], such that adolescents that generally have more negative affect also engage in more MVPA.

**Table 3:** MVPA: results for negative affect.

Fixed effects		95% CI for slope		
	Slope	Lower	Upper	*P*
Intercept	−0.07	−0.81	0.67	0.85
Time	0.00	−0.00	0.00	0.19
BP negative affect	0.09	0.014	0.16	**0.02**
WP negative affect	0.00	−0.02	0.03	0.87
Perceived competence	0.15	−0.00	0.29	0.05
Autonomous motivation	0.03	−0.14	0.20	0.70
Random effects	Estimate		SE	
Intercept	0.42		0.08	
Residual	5.88		0.11	

BP, between-person; WP, within-person; CI, confidence intervals.

Bold values indicate statistically significant results (*p* < 0.05).

#### 3.1.4 Fatigue

In the final model (Table 4), within-person fatigue, between-person fatigue, friend social support, amotivation, and controlled motivation no longer significantly predict engagement in MVPA. However, perceived competence remained a significant predictor (β = 0.23, *p* < 0.01) with 95% CI [0.09, 0.37], such that adolescents with higher perceived competence engage in more MVPA. There were also three significant interactions with within-person fatigue: friend social support (β = 0.05, *p* < 0.01) with 95% CI [0.02, 0.08], amotivation (β = 0.03, *p* < 0.05) with 95% CI [0.003, 0.06], and controlled motivation (β = −0.03, *p* < 0.01) with 95% CI [−0.05, −0.01].

**Table 4:** MVPA: results for fatigue.

Fixed effects		95% CI for slope		
	Slope	Lower	Upper	*P*
Intercept	0.41	−0.33	1.15	0.28
Time	0.00	−0.00	0.00	0.20
BP fatigue	0.06	−0.03	0.15	0.18
WP fatigue	−0.10	−0.20	0.00	0.06
Perceived competence	0.23	0.09	0.37	**0.001**
Friend social support	−0.14	−0.31	0.03	0.10
WP fatigue^*^friend social support	0.05	0.02	0.08	**0.003**
Amotivation	−0.12	−0.26	0.02	0.10
WP fatigue^*^Amotivation	0.03	0.00	0.06	**0.03**
Controlled motivation	−0.04	−0.16	0.08	0.55
WP fatigue^*^controlled motivation	−0.03	−0.05	−0.01	**0.006**
Random effects	Estimate		SE	
Intercept	0.43		0.08	
Residual	5.81		0.12	

BP, between-person; WP, within-person; CI, confidence intervals.

Bold values indicate statistically significant results (*p* < 0.05).

**Figure 2:**
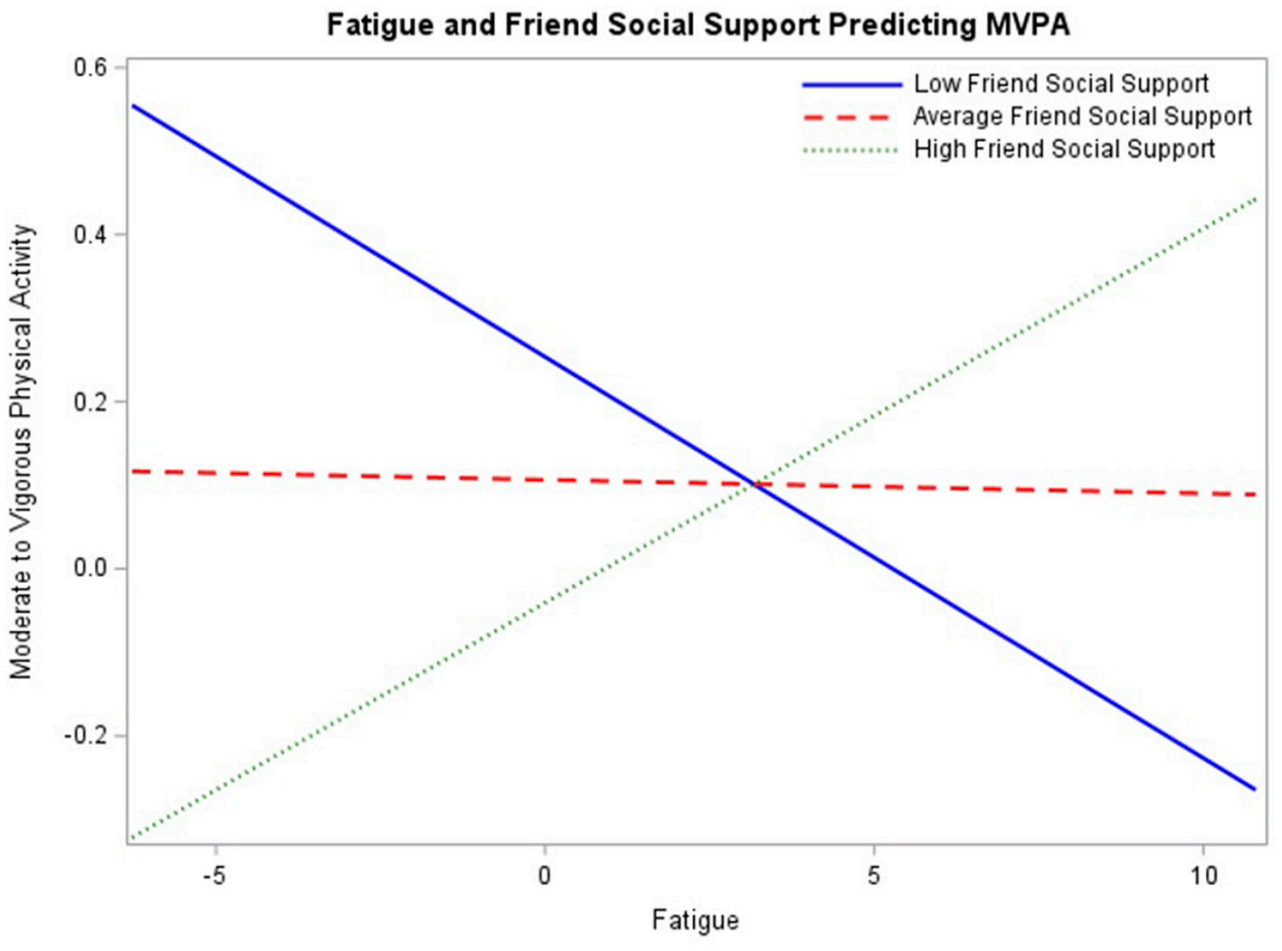
Interaction between fatigue and baseline friend social support predicting engagement in physical activity probed at the mean level of social support, as well as one standard deviation above and below the mean.

To assess the association between fatigue and engagement in MVPA throughout the range of baseline friend social support ratings, the interaction was probed at mean friend social support ratings as well as one standard deviation above and below the mean (Figure 2). Simple slopes at the conditional values were not significant, therefore only simple slopes beyond the regions of significance should be interpreted. Regions of significance testing

demonstrated interaction significance between fatigue and physical activity beyond −0.23 for low friend social support, −0.11 (*p* = 0.05) and 4.89 for high friend social support, 0.12 (*p* = 0.05), indicating that simple slopes are significant beyond these values. Due to the nature of these data, only the highest levels of fatigue fall within the region of significance, which suggests that the association between fatigue and MVPA is observed at the higher levels of fatigue and varies as a function of friend social support.

**Figure 3:**
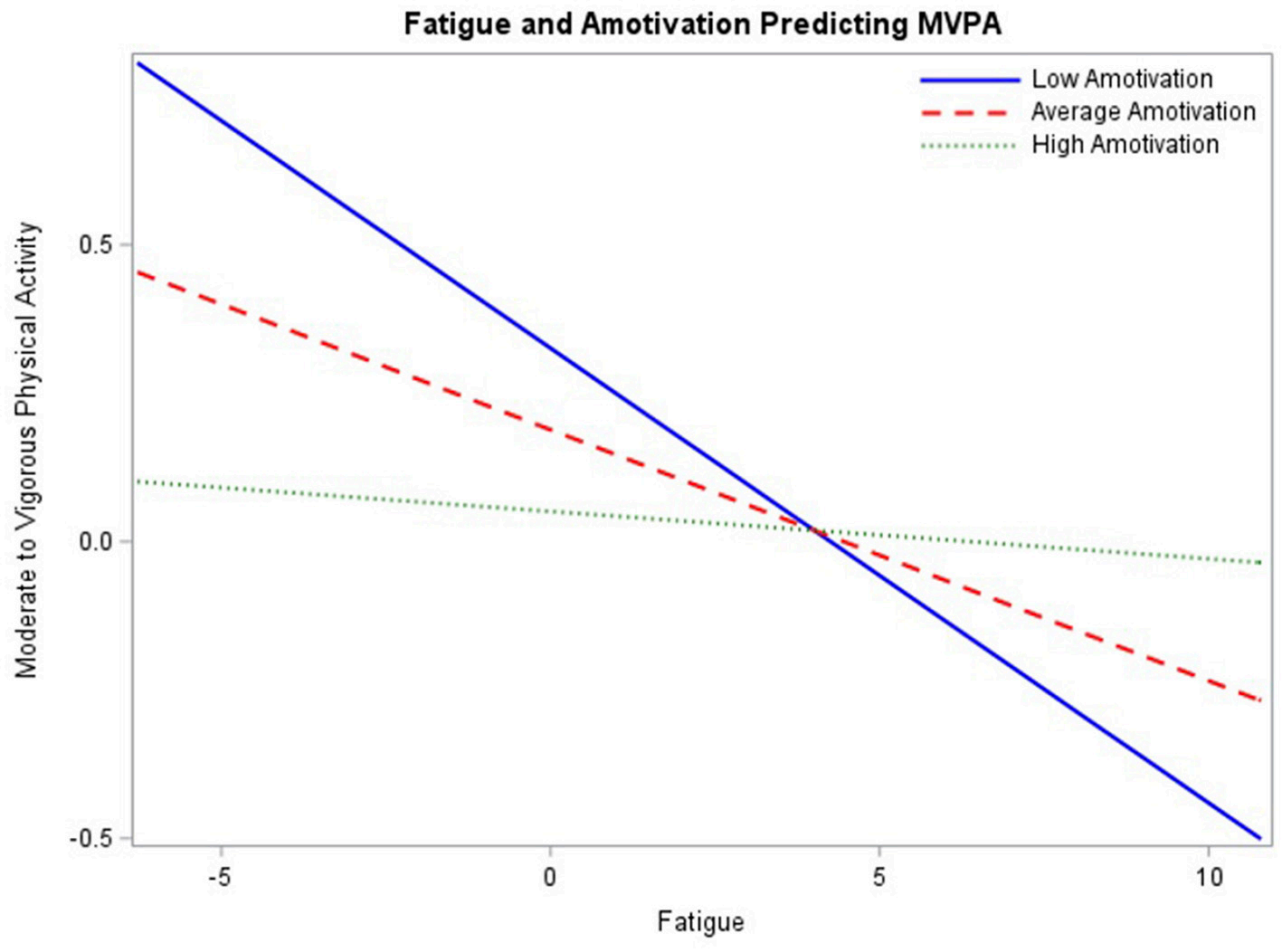
Interaction between fatigue and baseline amotivation ratings predicting engagement in physical activity probed at the mean level of amotivation, as well as one standard deviation above and below the mean.

To assess the association between fatigue and engagement in MVPA throughout the range of baseline amotivation ratings, the interaction was probed at mean amotivation ratings as well as one standard deviation above and below the mean (Figure 3). Simple slopes at the conditional values were not significant, therefore only simple slopes beyond the regions of significance should be interpreted. Regions of significance testing demonstrated interaction significance between fatigue and MVPA beyond −0.39 for low amotivation, 0.11 (*p* = 0.05) and 19.46 for high amotivation, 0.49 (*p* = 0.05), indicating that simple slopes are significant beyond these values. Notably, these values are not within the range of expected values for the data and therefore, will not be interpreted.

**Figure 4:**
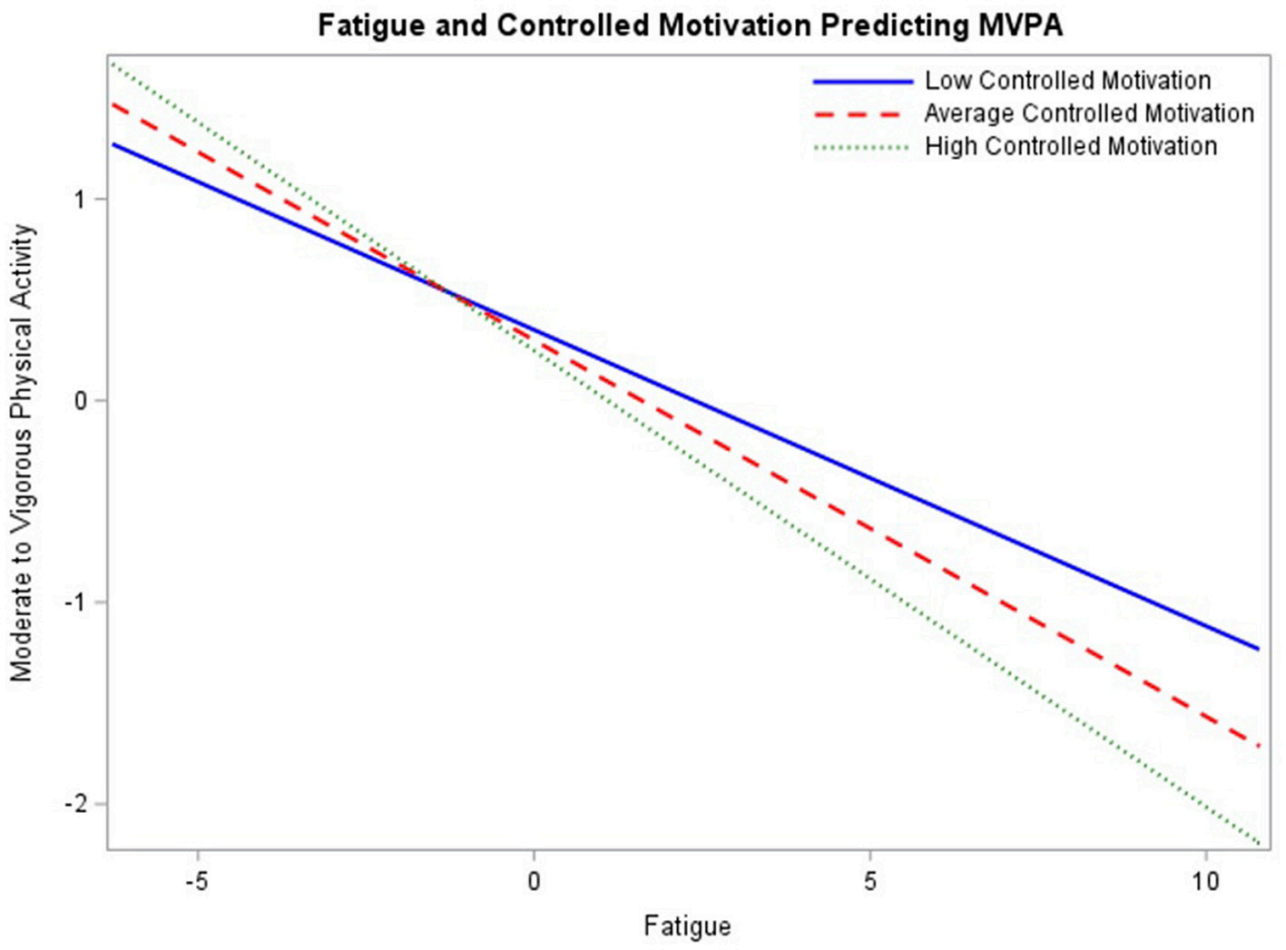
Interaction between fatigue and baseline controlled motivation ratings predicting engagement in physical activity probed at the mean level of controlled motivation, as well as one standard deviation above and below the mean.

To assess the association between fatigue and engagement in MVPA throughout the range of baseline ratings of controlled motivation, the interaction was probed at mean ratings as well as one standard deviation above and below the mean (Figure 4). At lower levels of controlled motivation, the simple slope between fatigue and engagement in MVPA, −0.15, was significant (*p* < 0.01). The simple slope was also significant at the mean of controlled motivation −0.19 (*p* < 0.001) and higher levels of controlled motivation, −0.23 (*p* < 0.001). Regions of significance testing demonstrated interaction significance between fatigue and MVPA beyond −19.53 for low controlled motivation and 0.16 for high controlled motivation, indicating that simple slopes are significant beyond these values. This suggests that higher fatigue is associated with lower MVPA across all levels of controlled motivation, with a stronger negative association at higher levels of controlled motivation.

#### 3.1.5 Energy

Results of the final model (Table 5) demonstrate that significant main effects are maintained for within-person energy (β = 0.04, *p* < 0.01) with 95% CI [0.01, 0.07] and perceived competence (β = 0.16, *p* < 0.01) with 95% CI [0.04, 0.28] as a predictor of engagement in MVPA. Between-person energy does not predict MVPA engagement in the final model. This model suggests that when an adolescent's energy is higher than normal, they're more likely to engage in MVPA and that those generally higher in perceived competence are also more likely to engage in MVPA.

**Table 5:** MVPA: results for energy.

Fixed effects		95% CI for slope		
	Slope	Lower	Upper	*P*
Intercept	0.15	−0.42	0.73	0.60
Time	0.00	−0.00	0.00	0.16
BP energy	0.01	−0.06	0.08	0.78
WP energy	0.04	0.01	0.07	**0.005**
Perceived competence	0.16	0.04	0.28	**0.009**
Random effects	Estimate		SE	
Intercept	0.45		0.08	
Residual	5.89		0.11	

BP, between-person; WP, within-person; CI, confidence intervals.

Bold values indicate statistically significant results (*p* < 0.05).

### 3.2 Sedentary behavior

#### 3.2.1 ICC

The results of the null model demonstrated that 8% of the variance in sedentary behavior was between-person. Importantly, this means that 92% of the variance can be accounted for by within-person moment-to-moment changes.

#### 3.2.2 Positive affect

Results of the final model (Table 6) demonstrated a significant main effect of within-person positive affect (β = −0.29, *p* < 0.001) with 95% CI [−0.42, −0.16] and a significant interaction between within-person positive affect and amotivation (β = 0.10, *p* < 0.01) with 95% CI [0.04, 0.16].

**Table 6:** Sedentary time: results for positive affect.

Fixed effects		95% CI for slope		
	Slope	Lower	Upper	*P*
Intercept	21.26	19.55	22.98	**<0.0001**
Time	−0.00	−0.02	0.01	0.42
BP positive affect	−0.01	−0.13	0.12	0.93
WP positive affect	−0.29	−0.42	−0.16	**<0.0001**
Amotivation	−0.09	−0.54	0.35	0.68
WP positive Affect^*^Amotivation	0.10	0.04	0.16	**0.002**
Random effects	Estimate		SE	
Intercept	5.12		1.25	
Time	0.00		0.00	
WP positive affect	0.03		0.02	
Residual	49.34		0.98	

BP, between-person; WP, within-person; CI, confidence intervals.

Bold values indicate statistically significant results (*p* < 0.05).

**Figure 5:**
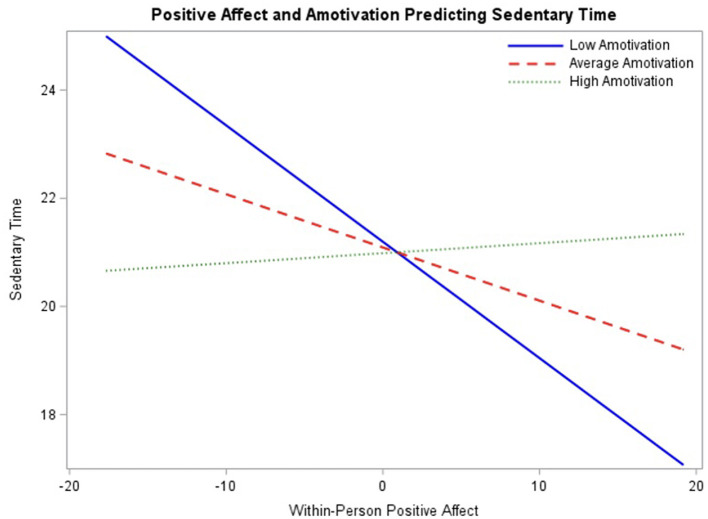
Interaction between positive affect and baseline amotivation ratings predicting engagement in sedentary behavior probed at the meal level of amotivation, as well as one standard deviation above and below the mean.

To assess the association between positive affect and sedentary behavior throughout the range of baseline amotivation ratings, the interaction was probed at mean amotivation ratings as well as one standard deviation above and below the mean (Figure 5). The simple slope between positive affect and sedentary behavior was significant at lower levels of amotivation, −0.22 (*p* < 0.001) and at the mean of amotivation −0.10 (*p* < 0.001), such that as within-person positive affect increases sedentary time decreases for those with baseline average and low amotivation. The simple slope was not significant at higher levels of amotivation, 0.02 (*p* > 0.05). Regions of significance testing demonstrated interaction significance between positive affect and sedentary behavior beyond 2.12 for low amotivation and 4.56 for high amotivation, indicating that simple slopes are significant beyond these values. This model suggests that generally, when an adolescent is experiencing more positive affect than typical, they're less likely to remain sedentary and that this is particularly true if they're average or low in amotivation.

#### 3.2.3 Negative affect

**Figure 6:**
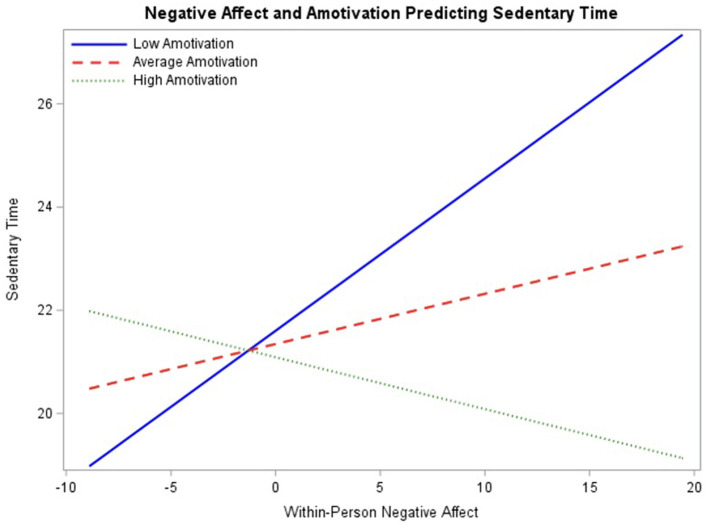
Interaction between negative affect and baseline amotivation ratings predicting engagement in sedentary behavior probed at the mean level of amotivation, as well as one standard deviation above and below the mean.

Results from the final model (Table 7) demonstrate a significant interaction between within-person negative affect and amotivation (β = −0.08, *p* < 0.05) with 95% CI [−0.14, −0.01]. To assess the association between negative affect and sedentary behavior throughout the range of baseline amotivation ratings, the interaction was probed at mean amotivation ratings as well as one standard deviation above and below the mean (Figure 6). The simple slope between negative affect and sedentary behavior was significant at higher levels of amotivation, −0.10 (*p* < 0.05), such that as within-person negative affect increases, sedentary time decreases for those with high amotivation. The simple slope was not significant at mean levels of amotivation, 0.10 (*p* > 0.05) or lower levels of amotivation, 0.30 (*p* > 0.05). Regions of significance testing demonstrated interaction significance between negative affect and sedentary behavior beyond −3.39 for low amotivation and 2.45 for high amotivation, indicating that simple slopes are significant beyond these values. This model suggests that when adolescents report higher-than-usual negative affect, those with higher levels of amotivation show decreases in sedentary behavior.

**Table 7:** Sedentary time: results for negative affect.

Fixed effects		95% CI for slope		
	Slope	Lower	Upper	*P*
Intercept	21.35	20.24	22.45	**<0.0001**
Time	−0.00	−0.01	0.01	0.43
BP negative affect	−0.06	−0.29	0.17	0.60
WP negative affect	0.10	−0.04	0.24	0.18
Amotivation	−0.10	−0.55	0.35	0.66
WP negative affect^*^amotivation	−0.08	−0.14	−0.01	**0.02**
Random effects	Estimate		SE	
Intercept	4.7910		1.1900	
Time	0.000737		0.000397	
Residual	49.9452		0.9795	

BP, between-person; WP, within-person; CI, confidence intervals.

Bold values indicate statistically significant results (*p* < 0.05).

#### 3.2.4 Fatigue

The final model did not demonstrate any significant predictors of sedentary time (Table 8).

**Table 8:** Sedentary time: results for fatigue.

Fixed effects		95% CI for slope		
	Slope	Lower	Upper	*P*
Intercept	21.26	20.33	22.19	**<0.0001**
Time	−0.00	−0.02	0.01	0.39
BP fatigue	−0.08	−0.37	0.21	0.60
WP Fatigue	0.07	−0.02	0.16	0.11
Random effects	Estimate		SE	
Intercept	4.7827		1.1944	
Time	0.000855		0.000429	
Residual	49.8245		0.9773	

BP, between-person; WP, within-person; CI, confidence intervals.

Bold values indicate statistically significant results (*p* < 0.05).

#### 3.2.5 Energy

In the final model (Table 9), within-person energy was the only significant predictor in the final model (β = −0.17, *p* < .001), such that as within person energy increased, sedentary time decreased.

**Table 9:** Sedentary time: results for energy.

Fixed effects		95% CI for slope		
	Slope	Lower	Upper	*P*
Intercept	21.08	20.06	22.11	**<0.0001**
Time	−0.00	−0.02	0.01	0.41
BP energy	−0.01	−0.22	0.20	0.95
WP energy	−0.17	−0.26	−0.09	**<0.0001**
Random effects	Estimate		SE	
Intercept	4.90		1.21	
Time	0.00		0.00	
Residual	49.71		0.98	

BP, between-person; WP, within-person; CI, confidence intervals.

Bold values indicate statistically significant results (*p* < 0.05).

### 3.3 Discussion

The goal of the project was to determine whether rational and automatic motivational processes interact in predicting physical activity (MVPA and sedentary behavior), with the aim of understanding whether effortful, conscious motivation may attenuate automatic vulnerabilities under certain conditions. Specifically, whether rational motivation can help adolescents overcome challenges to physical activity posed by automatic processes such as mood, fatigue, or energy levels. For example, we suspected that in moments when participants experience an unusual surge in fatigue, the attendant risk for low physical activity might be overcome by having a friend who supports exercise. Our findings suggest that a lack of rational motivation plays a crucial role: while positive mood generally encourages more MVPA, its effect is reduced when an adolescent has high levels of amotivation. In other words, a similar pattern emerged with sedentary behavior. When adolescents feel happier than usual, they are less likely to stay sedentary—again, unless amotivation is high. Interestingly, the relationship between negative mood and sedentary behavior was more complex. When amotivation is high, experiencing more negative emotions than usual is associated with decreased sedentary behavior. This suggests that individuals high in amotivation may be particularly responsive to momentary increases in negative affect.

Additionally, we found that adolescents that generally experience more negative affect also engage in more MVPA. Notably, this is a between-person association, as within-person negative affect and between-person negative affect have been found to have varying relationships with physical activity (Aggio et al., 2017). While past research would suggest that the direct effects of negative affect and physical activity were negatively associated with each other, the present study suggests that variability can exist between adolescents. Therefore, intervention targets that do not account for individual differences in response to automatic affective processes may inadequately support adolescents who do find themselves engaging in more MVPA despite elevated negative affect. Precise interventions that account for the observed between-person differences, and previous findings of the varying relationships (Aggio et al., 2017), can better support a wider range of adolescents.

Consistent with previous literature (Dunton et al., 2014), we found that when an adolescent's energy is higher than typical, they're more likely to engage in MVPA, suggesting an opportunity to support physical activity. Although sedentary behavior was associated with higher energy than typical in an adolescent, this marks a contrast with past research (Cushing et al., 2017), indicating a more complex relationship between energy and physical activity. This relationship warrants further investigation into how sedentary behavior is associated with feelings of energy and subsequent physical activity, such as the potential feeling of restfulness being a moderator of engagement. Adolescent high friend social support may protect against fatigue, and even encourage increased engagement in physical activity (Duncan et al., 2005). For example, an adolescent who maintains a strong friend group can be encouraged to engage in more physical activity through shared activities and general fitness support. We also found that adolescents with higher perceived competence are also more likely to engage in physical activity. In other words, adolescents who believe they are self-motivated and believe in their ability to engage in physical activity translates to true engagement in physical activity. Physical activity interventions could use this phenomenon to support increased engagement, drawing on an adolescent's own perceived competence, motivation, and behavior to improve activity goals.

Notably, there are other models for understanding the role of affect in predicting physical activity, including the Affect and Health Behavior Framework that breaks affective constructs into (1) affect response (e.g., feeling in response to engaging in physical activity) (2) incidental affect (e.g., affect unrelated to a behavior), (3) affect processing (i.e., how an individual perceives, interprets, and responds to an emotion), and (4) affectively charged motivational states (i.e., motivation rooted in previous experience) (Stevens et al., 2020). This study focused specifically on incidental affect, as we measured affect independent of physical activity engagement. Affective feelings separate from outside stimuli, emotions, and motivation provide us with a more accurate representation of affect as it pertains to moment-to-moment feelings. In other words, by removing dependency on other factors we understand affect on more granular level. It is important to recognize that our findings may differ when examining other facets of affect. Additionally, we accounted for individual differences by allowing for unique slopes in our model where appropriate (i.e., if model fit improved). This approach contrasts with much of the previous research, which often assumes a shared slope across all individuals, potentially overlooking important variations that could influence the conclusions. In the development of physical activity interventions for adolescents, one cannot make the assumption that the observed variables are consistent between individuals. For example, each adolescent may react differently to elevated affective feelings. By allowing for unique slopes, we better encompass the unique variation of each adolescent. Physical activity interventions should follow a similar approach, where recognizing and accounting for individual differences is paramount in meeting the personal intervention goals for each adolescent.

#### 3.3.1 Limitations and future directions

Further research is needed to understand the interplay between rational and automatic motives and how to effectively intervene to promote MVPA and decrease sedentary time. Participants reported affect, energy, and fatigue at the same time each day, therefore it is important to conduct additional studies to explore how this relationship might differ when reporting is randomized. Additionally, our predominantly White sample may limit the generalizability of findings to more diverse populations. Additionally, the observational nature of the current study also limits the ability to draw causal inferences regarding the relationships between rational and automatic processes and behavioral outcomes. Further, although ecological momentary assessment allows for the capture of real-time processes, approximately 50% of EMA reports were missing, which may impact internal validity and the representativeness of momentary associations. From an analytic perspective, the present study modeled each automatic process separately and evaluated cognitive predictors independently, retaining predictors based on statistical significance and model fit. While this approach allowed us to isolate specific associations and reduce model complexity, it may limit direct comparability across models and does not fully account for potential suppression effects that could emerge when multiple predictors are considered simultaneously.

Future research should examine these processes within more fully specified models to better capture the joint and potentially interdependent effects of rational and automatic motivational factors. Increasing sample diversity will also be critical to improve generalizability, as will efforts to reduce missingness in EMA data to strengthen internal validity. Intervention development may benefit from identifying moments of heightened vulnerability (e.g., low energy or negative affect) and targeting rational motivational processes, such as enhancing perceived competence or social support, to support adaptive behavioral responses in real time. Additionally, future studies should systematically evaluate different temporal windows (e.g., shorter or longer intervals surrounding EMA prompts) to determine how the choice of assessment window influences the observed associations between momentary affect states and physical activity. Findings from this study highlight the intertwined nature of rational and automatic motives in shaping physical activity. Importantly, this means that interventions aimed at promoting physical activity should consider both motivation systems as join targets rather than in isolation.

## Data Availability

The raw data supporting the conclusions of this article will be made available by the authors, without undue reservation.
